# An Atypical Presentation of Fabry Disease in a Patient With Nephrotic Syndrome: A Case Report

**DOI:** 10.7759/cureus.63661

**Published:** 2024-07-02

**Authors:** Manasi Harale, Arun B Oommen, Mayank Mundada, Ahsan A Faruqi, Shivraj Patil

**Affiliations:** 1 General Medicine, Dr. D. Y. Patil Medical College, Hospital and Research Centre, Dr. D. Y. Patil Vidyapeeth, Pune (Deemed to be University), Pune, IND

**Keywords:** clinical nephrology, genetic renal diseases, general internal medicine, internal medicine, idiopathic nephrotic syndrome, fabry's disease

## Abstract

Fabry disease is a rare X-linked lysosomal storage disorder that leads to the accumulation of globotriaosylceramide (Gb3) across various tissues, stemming from a deficiency in alpha-galactosidase A (GLA). This condition is characterized by a spectrum of clinical manifestations that can significantly complicate diagnosis. Classical symptoms typically include neuropathic pain, angiokeratomas, and significant involvement of the renal and cardiac systems. However, atypical presentations may obscure the underlying diagnosis, emphasizing the importance of maintaining a high level of clinical suspicion. This case report details the diagnostic journey of a 24-year-old female who initially presented with nephrotic syndrome, a presentation not commonly associated with Fabry disease. Subsequent genetic testing revealed a pathogenic variant in the GLA gene, confirming Fabry disease and highlighting the critical need for genetic analysis in cases of unexplained renal pathology. This case underscores the variability of Fabry disease presentations and the pivotal role of comprehensive diagnostic strategies in uncovering this complex disorder.

## Introduction

Fabry disease is often underdiagnosed due to its wide phenotypic variability and overlap with more common conditions. Classic symptoms include acroparesthesia, angiokeratomas, hypohidrosis, and corneal verticillata, which are typically present in childhood or adolescence. The worldwide prevalence of Fabry disease is estimated to range from 1:40,000 to 1:170,000, with recent newborn screening studies suggesting a higher prevalence of approximately 1:1250. This variability in prevalence highlights underdiagnosis and the importance of awareness for accurate diagnosis and management [1,2]. However, atypical presentations, especially in adult patients with isolated renal or cardiac symptoms, pose significant diagnostic challenges [3,4]. Alpha-galactosidase-A (GLA) enzyme deficiency, which causes globotriaosylceramide (Gb3) to accumulate in different tissues, is the cause of Fabry disease. This disease is caused by mutations in the GLA gene [5]. Pathophysiologically, this accumulation causes cellular dysfunction and damage, particularly affecting the kidneys, heart, and nervous system [6,7]. Diagnostic methods include enzyme assays, genetic testing, and imaging studies [8,9]. Differential diagnoses can include other lysosomal storage disorders, chronic kidney disease of unknown origin, and various cardiovascular conditions [10]. Treatment options for Fabry disease include enzyme replacement therapy (ERT), chaperone therapy, and emerging gene therapies [6-8].

## Case presentation

A 24-year-old female patient arrived at the hospital exhibiting significant edema of the lower extremities, frequent nocturia, and excessive thirst. Her vital signs showed a temperature of 98.6°F, a heart rate of 88 beats per minute, a respiration rate of 16 breaths per minute, and a blood pressure of 180/110 mmHg. On physical examination, no angiokeratomas or corneal abnormalities were noted. The cardiovascular examination revealed elevated blood pressure, and an echocardiogram showed left ventricular hypertrophy.

Routine lab tests revealed hypoalbuminemia and hypercholesterolemia, with serum albumin at 2.8 g/dL, total cholesterol at 302 mg/dL, and elevated blood creatinine levels at 1.5 mg/dL. All other tests, including serum electrolytes, liver function tests (LFTs), and a complete blood count (CBC), were normal. Complement levels (C3, C4) were within normal ranges, and autoimmune tests, including cytoplasmic antineutrophil cytoplasmic antibody (c-ANCA) and perinuclear antineutrophil cytoplasmic antibody (p-ANCA), were negative (Table 1).

**Table 1:** Laboratory test results for autoimmune and complement levels EIA: Enzyme-linked immunosorbent assay; p-ANCA: Perinuclear antineutrophil cytoplasmic antibody; c-ANCA: Cytoplasmic antineutrophil cytoplasmic antibody; MPO: Myeloperoxidase; PR3: Proteinase 3

Test description	Observed value	Biological reference interval
ANCA-MPO (p-ANCA) - serum by EIA	Negative (1.395)	Negative: <20 RU/mL, positive: >=20 RU/mL
ANCA-PR3 (c-ANCA) - serum by EIA	Negative (<2)	Negative: <20 RU/mL, positive: >=20 RU/mL
Complement 4 (C4) - serum by nephelometry	30.90 mg/dL	10-40 mg/dL
Complement 3 (C3) - serum by nephelometry	131.00 mg/dL	90-180 mg/dL

**Figure 1:**
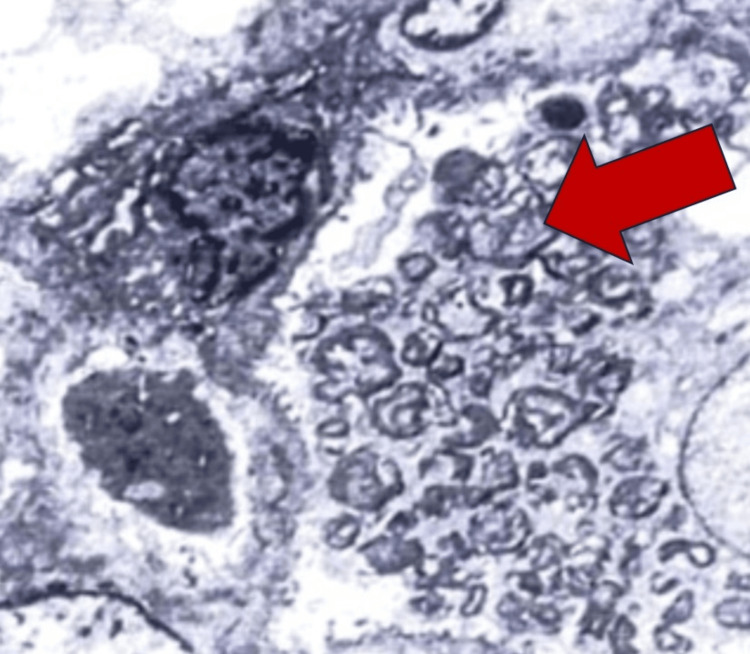
Electron microscopy showing zebra bodies indicated by a red arrow within the visceral epithelial cell cytoplasm

An ultrasound (USG) of the kidneys indicated normal-sized kidneys with increased cortical echogenicity. Due to the unexplained renal dysfunction, along with significant proteinuria, a kidney biopsy was performed. Histopathological findings from light microscopy revealed global and segmental glomerulosclerosis with prominent vacuolization of the podocytes. Electron microscopy (Figure 1) demonstrated osmiophilic lamellated structures known as zebra bodies within the visceral epithelial cell cytoplasm, suggesting the diagnosis of Fabry disease. To confirm this, specific biochemical tests were conducted. Plasma lyso-Gb3 levels were found to be elevated at 45 ng/mL (normal range: <1.8 ng/mL), and GLA activity in leukocytes was markedly reduced, measured at 3 nmol/hr/mg protein (normal range: 40-200 nmol/hr/mg protein). Given the results of the biopsy and biochemical tests, a diagnosis of Fabry disease was established.

Genetic testing confirmed the diagnosis by identifying a pathogenic variant in the GLA gene (c.1088G>A, p.Arg363His) associated with Fabry disease (Table 2).

**Table 2:** Whole exome sequencing testing confirmed the diagnosis by identifying a pathogenic variant in the GLA gene (c.1088G>A, p.Arg363His) associated with Fabry disease GLA: Alpha-galactosidase A

Test	Inference	Normal value
Whole exome sequencing	Pathogenic variant in GLA gene (c.1088G>A, p.Arg363His) confirmed, associated with Fabry disease	No pathogenic variant

Given the diagnosis of Fabry disease, a comprehensive treatment plan was initiated. The patient was started on telmisartan 40 mg once daily to manage proteinuria and control blood pressure. Dietary modifications such as salt restriction and controlled protein intake were advised to reduce the burden on the kidneys and manage edema [6-8]. The initial follow-up after two months showed a significant decrease in proteinuria and a reduction in pedal edema, with better-controlled blood pressure. Regular follow-ups every three to six months were planned to monitor kidney function, proteinuria levels, and blood pressure. Long-term management included exploring financial assistance for ERT since the patient could not afford it and providing patient education about Fabry disease, adherence to the treatment plan, and genetic counseling. Referrals to a nephrologist, cardiologist, and genetic counselor were made to ensure a multidisciplinary approach to managing the patient's condition and improving her quality of life.

## Discussion

This case highlights the phenotypic heterogeneity of Fabry disease. The patient's presentation with nephrotic syndrome, significant proteinuria, and the absence of classic symptoms initially diverted attention from Fabry disease. The identification of a GLA gene mutation through whole exome sequencing (WES) was crucial in diagnosing this atypical presentation. WES involves sequencing all the protein-coding regions of genes in a genome, capturing and sequencing these regions, and then using bioinformatic tools to analyze the data and identify genetic variants. This comprehensive approach allows for the detection of mutations that may not be evident through traditional diagnostic methods. Early diagnosis and treatment are vital to prevent irreversible organ damage and improve patient outcomes [3,4].

Nephrotic syndrome as a manifestation of Fabry disease is relatively rare. However, there are documented cases where nephrotic syndrome was the primary presenting symptom. For instance, a study reported that a 67-year-old patient with Fabry disease developed nephrotic syndrome and experienced a significant reduction in proteinuria following immunosuppressive therapy, highlighting the potential for atypical renal presentations in Fabry disease [5]. In Fabry disease patients, ERT has also been demonstrated to stabilize renal function and lower proteinuria, highlighting the significance of early treatment intervention [11].

The clinical symptoms, in this case, included pallor, hypertension, and palpitations, which led to the suspicion of Fabry disease or nephrotic syndrome. Confirming the diagnosis of Fabry disease, WES revealed a pathogenic mutation responsible for the phenotype. This aligns with other reported cases where genetic testing played a crucial role in diagnosing Fabry disease in patients with atypical presentations [7,8]. For example, in another study, a patient presented with proteinuria and ventricular septal thickening, and subsequent genetic testing confirmed Fabry disease. The patient responded well to ERT, further supporting the benefits of early genetic diagnosis and treatment [7,11].

## Conclusions

When making a differential diagnosis for nephrotic syndrome, particularly in younger individuals with unexplained renal failure, Fabry disease should be taken into account. Advanced genetic testing and biomarker analysis are essential for diagnosing atypical cases and facilitating timely intervention and management. Medical professionals and students should be vigilant for Fabry disease in patients presenting with nephrotic syndrome. Comprehensive testing, including histopathological examination, enzyme assays, and genetic testing, is crucial for confirmation. Genetic counseling and family testing are recommended to assess the risk to relatives and inform future medical decisions. It is important to note that, although Fabry disease is X-linked, females may develop manifestations similar to males. A multidisciplinary approach, collaborating with pathologists, geneticists, and nephrologists, is essential for accurate diagnosis and effective management.

## References

[1] Germain DP (2002). Fabry’s disease (alpha-galactosidase-A deficiency): recent therapeutic innovations (Article in French). J Soc Biol.

[2] Monte MA, Veroux M, Rodolico MS (2022). Fabry’s disease: the utility of a multidisciplinary screening approach. Life (Basel).

[3] Desnick RJ, Allen KY, Desnick SJ, Raman MK, Bernlohr RW, Krivit W (1973). Fabry's disease: enzymatic diagnosis of hemizygotes and heterozygotes. Alpha-galactosidase activities in plasma, serum, urine, and leukocytes. J Lab Clin Med.

[4] Eng CM, Fletcher J, Wilcox WR (2007). Fabry disease: baseline medical characteristics of a cohort of 1765 males and females in the Fabry Registry. J Inherit Metab Dis.

[5] Ortiz A, Oliveira JP, Waldek S, Warnock DG, Cianciaruso B, Wanner C (2008). Nephropathy in males and females with Fabry disease: cross-sectional description of patients before treatment with enzyme replacement therapy. Nephrol Dial Transplant.

[6] Brady RO, Schiffmann R (2000). Clinical features of and recent advances in therapy for Fabry disease. JAMA.

[7] Branton MH, Schiffmann R, Sabnis SG (2002). Natural history of Fabry renal disease: influence of alpha-galactosidase A activity and genetic mutations on clinical course. Medicine (Baltimore).

[8] Biegstraaten M, Arngrímsson R, Barbey F (2015). Recommendations for initiation and cessation of enzyme replacement therapy in patients with Fabry disease: the European Fabry Working Group consensus document. Orphanet J Rare Dis.

[9] Michaud M, Mauhin W, Belmatoug N (2020). When and how to diagnose Fabry disease in clinical pratice. Am J Med Sci.

[10] Chen Z, Yin B, Jiao J, Ye T (2024). Case report: enzyme replacement therapy for Fabry disease presenting with proteinuria and ventricular septal thickening. BMC Nephrol.

[11] Schiffmann R, Kopp JB, Austin HA (2001). Enzyme replacement therapy in Fabry disease: a randomized controlled trial. JAMA.

